# FINANCIAL HARDSHIPS AND DEPRESSIVE SYMPTOMS DURING COVID: THE MODERATING ROLE OF RESILIENCE

**DOI:** 10.1093/geroni/igac059.2984

**Published:** 2022-12-20

**Authors:** Brooke Helppie-McFall, Dawn Carr, Miles Taylor, Amanda Sonnega

**Affiliations:** University of Michigan, Ann Arbor, Michigan, United States; Florida State University, Tallahassee, Florida, United States; Florida State University, Tallahassee, Florida, United States; University of Michigan, Ann Arbor, Michigan, United States

## Abstract

Financial hardships during COVID (FHDC) are a particularly salient stressor that older people faced during the pandemic. Financial stress is associated with increased depressive symptoms and may be especially consequential to mental health among older people who have fewer resources (i.e., financially vulnerable). Recent evidence shows that psychological resilience has important protective effects for mental health among older adults who experience major stressors. This study, based on the recently released full 2020 core wave of the Health and Retirement Study, examines the association between having experienced one or more major financial setbacks following the start of the pandemic and depressive symptoms. We evaluate the consequences of FHDC for mental health, how FHDC are associated with previous financial vulnerability, and the role of psychological resilience in shaping the effects of FHDC. Results show that having experienced FHDC is associated with an increase in depressive symptoms. In addition, reporting financial vulnerabilities four years prior to the pandemic was also associated with increased depressive symptoms. Finally, psychological resilience was associated with a significant, protective effect on depressive symptoms, and moderated the consequences of FHDC. Specifically, we find that those who had FHDC and had average or below average resilience experienced significant increases in depressive symptoms, but those with above average resilience did not experience increases in depressive symptoms despite having FHDC, accounting for the consequences of previous financial vulnerabilities. These results suggest that psychological resilience has potential to be a protective resource for mental health consequences of financial stress among older adults.

